# Pseudo-Core-Shell Permalloy (Supermalloy)@ZnFe_2_O_4_ Powders and Spark Plasma Sintered Compacts Based on Mechanically Alloyed Powders

**DOI:** 10.3390/ma17164139

**Published:** 2024-08-21

**Authors:** Traian Florin Marinca, Loredana Cotojman, Florin Popa, Bogdan Viorel Neamțu, Călin-Virgiliu Prică, Ionel Chicinaș

**Affiliations:** Materials Science and Engineering Department, Technical University of Cluj-Napoca, 400641 Cluj-Napoca, Romania; traian.marinca@stm.utcluj.ro (T.F.M.); cotojman.loredana@yahoo.com (L.C.); florin.popa@stm.utcluj.ro (F.P.); bogdan.neamtu@stm.utcluj.ro (B.V.N.); calin.prica@stm.utcluj.ro (C.-V.P.)

**Keywords:** pseudo-core-shell particles, soft magnetic composites, spark plasma sintering

## Abstract

Soft magnetic composite cores were produced by spark plasma sintering (SPS) from Ni_3_Fe@ZnFe_2_O_4_ and NiFeMo@ZnFe_2_O_4_ pseudo-core-shell powders. In the Fe-Ni alloys@ZnFe_2_O_4_ pseudo-core-shell composite powders, the core is a large nanocrystalline Permalloy or Supermalloy particle obtained by mechanical alloying, and the shell is a pseudo continuous layer of Zn ferrite particles. The pseudo-core-shell powders have been compacted by SPS at temperatures between 500–700 °C, with a holding time of 0 min. Several techniques were used for the characterisation of the powders and sintered compacts: X-ray diffraction, scanning electron microscopy, energy dispersive X-ray spectroscopy, magnetic hysteresis measurements (DC and AC), and electrical resistivity. The electrical resistivity is stabilised at values of about 7 × 10^−3^ Ω·m for sintering temperatures between 600–700 °C and this value is three orders of magnitude higher than the electrical resistivity of sintered Fe compacts. The best relative initial permeability was obtained for the Supermalloy/ZnFe_2_O_4_ composite compacts sintered at 600 °C, which decreases linearly for the entire frequency range studied, from around 95 to 50. At a frequency of 2000 Hz, the power losses are smaller than 1.5 W/kg. At a frequency of 10 kHz, the power losses are larger, but they remain at a reduced level. In the case of Supermalloy/ZnFe_2_O_4_ composite compact SPS-ed at 700 °C, the specific power losses are even lower than 5 W/kg. The power losses’ decomposition proved that intra-particle losses are the main type of losses.

## 1. Introduction

Soft magnetic composite materials (SMCs) have been of great research and application interest for several decades. These types of magnetic materials hold promise for better soft magnetic materials that can operate at high frequencies (implying high electrical resistivity) with very good magnetic properties such as inductance and permeability. The usual way to produce such a composite consists of coating the ferromagnetic particles by a dielectric thin layer and then compacting the particles by powder metallurgy techniques [1,2,3,4,5,6,7].

Soft magnetic composite materials (SMC) were produced by powder metallurgy from soft ferromagnetic powders, which are pressed together with an organic/inorganic dielectric. Several powders like Fe, Fe_3_P, Fe-Ni, Fe-Si, Fe-Si-Al, or Mo-Permalloy were used as ferromagnetic phase [8,9,10,11]. As a dielectric binder, it can be used as either an organic dielectric (epoxy resin, elastomers, etc.) or an inorganic binder (phosphates, oxides, glass, and so on) [10].

One of the new classes of soft magnetic materials is alloy/ferrite composites [12]. The aim of producing soft magnetic composites from alloy and ferrite is to obtain a material with an electrical resistivity closer to that of ferrite and a magnetic induction closer to that of the alloy. The use of soft magnetic ferrite as a coating agent can also improve the application in AC of Fe-based SMCs because the coating layer has high electrical resistivity [13].

In addition to its interesting properties, this type of composite material is very attractive due to the low cost of iron and hematite that are used as basic raw materials [14,15]. Very important is the insulating phase, which determines the mechanical properties, density, electrical resistivity, and all magnetic properties of SMCs. MnZn, NiZn, and Ni ferrite have high resistivities and also good magnetic properties. The most obvious advantage of these coatings is the reduced number of air gaps between the metal powder particles; these air gaps lead to effectively reducing the magnetic material’s saturation magnetisation and permeability. In addition, ferrite is a soft magnetic material that can be used at higher temperatures and frequencies [16,17].

An interesting approach to obtain SMCs can be to use the ferromagnetic particles having a dielectric layer from ferrites and compacting by spark plasma sintering (SPS). The SPS is one of the electric current-assisted sintering techniques. This technique involves the application of a DC current pulse through the pressing system, consisting of graphite die, which contains the powder sample, and graphite punches, simultaneously with a pressure applied. The advantages of SPS are the lower sintering temperature and time compared to the classical sintering [18]. The densification of the alloy/ferrite composite powders by SPS can be difficult due to the possibility to have a reaction between the composite phases. To avoid this reaction, the sintering parameters must be chosen carefully. The sintering time in SPS technique is very short and the sintering temperature is lower than in classical sintering. These parameters help to avoid reactions between the composite particle phases during the sintering process [17,18,19,20,21].

The paper presents results concerning producing and characterising Permalloy (Supermalloy)@ZnFe_2_O_4_ pseudo-core-shell particles and soft magnetic composite compacts from as-obtained powders produced via the spark plasma sintering densification technique. Our approach is to obtain soft magnetic composites of a Ni-based alloy in a zinc ferrite (ZnFe_2_O_4_) resistive matrix. The zinc ferrite shell of the pseudo-core-shell particles will create a network structure that will assure an enhanced electrical resistivity after sintering in the Ni_3_Fe/ZnFe_2_O_4_ and Supermalloy/ZnFe_2_O_4_ type soft magnetic composite. This procedure is a new and simple route which involves an accessible way to cover the Ni-based ferromagnetic particles with a thin layer of zinc ferrite particles, and it uses a low-energy compaction technique. The SMC with ferromagnetic Ni-based particles and ferrite as a dielectric has been obtained by SPS until now, but such a combination of precursors and production itinerary to our knowledge has not been used before.

## 2. Materials and Methods

Nanocrystalline soft magnetic Permalloy (Ni_3_Fe) and Supermalloy (79Ni16Fe5Mo, wt.%) were achieved by mechanical alloying using elemental powders: Fe (NC 100.24, purity 99.85%, produced by Höganäs AB, Höganäs, Sweden), Ni carbonyl 123 (purity 99.9%, Alpha Aesar, Kandel, Germany), and Mo (purity 99.95%, Alpha Aesar, Kandel, Germany) obtained by chemical reduction. Mechanical alloying was performed for up to 12 h in a planetary ball mill produced by Fritsch, model Pulverisette 6 (Idar-Oberstein, Germany). The alloying/milling process was performed in a high-purity argon atmosphere using a 500 mL vial and 14 mm diameter balls. The 12 h mechanically alloyed powder was annealed at 350 °C for 4 h to remove the internal stresses induced by the milling process and to ensure that the solid-state reaction to form Ni_3_Fe intermetallic compound as a single phase was finished.

Nanocrystalline Ni_3_Fe and Supermalloy powders obtained by mechanical alloying after 12 h of milling and ZnFe_2_O_4_ powders have been used to obtain pseudo-core-shell powders, which consist of a core of very large Ni_3_Fe or Supermalloy particles covered by a discontinuous layer (shell) of Zn ferrite. The ferrite particles have the chemical formula ZnFe_2_O_4_ and were supplied by Alfa Aesar of Kandel, Germany. The Zn ferrite particle size is less than 10 µm. The amount of 5 wt.% of zinc ferrite was calculated to result in a volume percentage of ferrite in the mixture of about 10 vol.%.

The resulting mixtures were homogenised for 15 min with a Turbula-type spatial homogeniser to ensure the most uniform distribution of particles. Due to the difference in particle size between Ni_3_Fe and Supermalloy powders on one side and Zn ferrite powder (much finer) on the other side, the result was alloy powder particles coated with a fine layer of ferrite particles.

The pseudo-core-shell Permalloy (Supermalloy)@ZnFe_2_O_4_ composite particles were subjected to compaction by spark plasma sintering (SPS), using home-made equipment [22,23]. The SPS process was performed in a graphite die by applying a pulsating current to the powders of maximum intensity 2500 A in an argon atmosphere and the sintering chamber was cooled by water pipes. The sintering process was performed in an argon atmosphere to prevent additional oxygen in the samples and to protect the die and punches. A constant pressure of 30 MPa was kept during the sintering (heating and cooling) cycle. In the heating sequence, the temperature heating rate was approximately 5 °C/s. The powders were sintered at temperatures of 500, 600, 650, and 700 °C, and the holding time at the sintering temperature was zero minutes. That means that the samples were only heated to the sintering temperature, and then the current was cut followed by the cooling process down to room temperature. We used a holding time of 0 min to limit the diffusion time, to limit the reaction between metallic phases and ferrite. The sintering temperature was measured by a K type thermocouple, which pass through the graphite die very close to the sample. It is about 1 mm; therefore, the temperature reached by the sample is with good approximation the one indicated by thermocouple. The cylindrical-shaped samples produced by SPS were drilled for toroidal shaping. Before drilling, the sintered compacts were metallographically conditioned using silicon carbide type sandpaper and alumina solution. The so obtained toroidal-shaped samples were wired with 0.5 mm (for primary coil) and 0.35 mm (for secondary coil) copper wire.

The formation of Permalloy and Supermalloy alloys by mechanical alloying, as well as the phase retention in sintered compacts, were studied by X-ray diffraction using the INEL 3000 Equinox diffractometer (INEL, Artenay, France), operating with radiation CoKα (1.79026 Å) in the angular range 2 θ = 20–110 degrees. The INEL 3000 Equinox diffractometer works in reflection mode and has a curved detector that simultaneously covers 90 degrees. The mean crystallite size was calculated for the annealed samples by the Scherrer method from the full-width-at-half-maximum (FWHM) of the diffraction peaks [24]. The resolution of the diffractometer was determined using a reference Ni sample. The morphology of the powders and the microstructure of the sintered compacts were investigated by scanning electron microscopy (SEM). A JEOL-JSM 5600-LV (Tokyo, Japan) was used, working with the secondary electron signal. The JEOL JSM 5600-LV microscope is equipped with an Oxford Instruments EDX detector, model ULTIMMAX65 (High Wycombe, UK) and Aztec analysis software, Version 4.2. X-ray microanalysis was used to determine the chemical homogeneity of mechanically alloyed powders as well as local chemical analysis and phase identification (by chemical element distribution maps) in composite powders and compacts. The density of composite compacts was obtained by determining the mass and geometric dimensions of cylindrical compacts (diameter and height). The samples’ mass was measured at least 5 times, as well as all the physical dimensions. The electrical characteristics were investigated with an electrical resistivity measuring system, which involves determining the electrical resistance on sintered compacts fixed between 2 flat contacts applied to the bases of cylindrical compacts. The SPS-ed composite compacts were magnetically characterised in DC and AC regimes. A computer-controlled hysteresisgraph Magnet-Physik Dr. Steingroever GmbH, Rema-graph–Remacomp C–705 model (Cologne, Germany) was used to register hysteresis curves. The AC tested frequency ranged between 50–10,000 Hz, and the induction level was seated at 0.01 T.

## 3. Results and Discussions

### 3.1. Permalloy, Supermalloy, and Permalloy (Supermalloy)@ZnFe_2_O_4_ Powders

The obtaining of Permalloy (Ni_3_Fe) and Supermalloy (79Ni16Fe5Mo, wt.%) by mechanical alloying used in this study was detailed in our previous papers [25,26]. It was proved that the Ni_3_Fe phase is obtained gradually by up to 8 h of milling. For up to 2 h of milling, the (200) and (211) diffraction peaks of Fe are present in the XRD patterns. Also, the asymmetry of the (200) and (220) diffraction peaks of Ni are visible at this milling time, and this asymmetry proves the presence of some Ni_3_Fe germs in powder. The milling was continued up to 12 h to ensure the solid-state reaction was completed in the entire sample volume, knowing that X-ray diffraction could not reveal the small residual phases [23].

Concerning the Supermalloy powders, it was shown in Reference [24] that after 8 h of milling, the milled sample contains only the Supermalloy phase. The progressive Supermalloy formation during milling is a result of the energy transfer from the milling bodies to the powders. Thus, changes in the XRD patterns show the progressive vanishing of the elemental powder (Fe and Mo) Bragg peaks and the progressive shift of the Ni Bragg peaks to lower diffraction angles, at the positions of the Supermalloy Bragg peaks [24]. 

The mean crystallite size of Ni_3_Fe and Supermalloy powders is around 16–18 ± 2 nm. The other details about obtaining Permalloy and Supermalloy powders by mechanical alloying are presented in our previous papers in this field [25,26,27]. 

**Figure 1:**
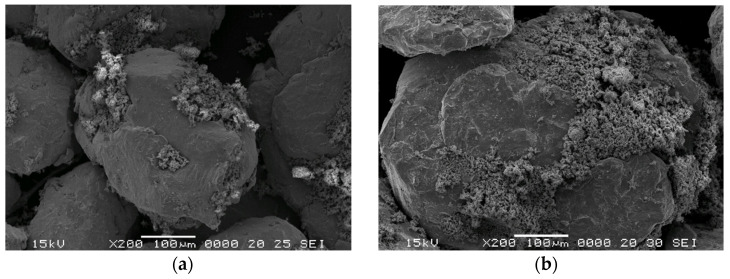
Morphology of powders homogenised for 20 min: (**a**) Ni_3_Fe@ZnFe_2_O_4_ and (**b**) 79Ni16Fe5Mo@ZnFe_2_O_4_.

Nanocrystalline Ni_3_Fe and Supermalloy powders obtained by mechanical alloying after 12 h of milling and ZnFe_2_O_4_ powders have been used to obtain pseudo-core-shell powders. By homogenisation, the very small particles of Zn ferrite cover more large particles of Ni_3_Fe and Supermalloy. The result is pseudo-core-shell particles, which consist of a core of very large Ni_3_Fe or Supermalloy particles covered by a discontinuous layer (shell) of fine Zn ferrite, Figure 1. The adherence of the fine zinc ferrite particles on the surface of Permalloy/Supermalloy larger particles is enhanced by the roughness surface provided upon producing the particles by mechanical alloying. All the pseudo-core-shell particles have an irregular shape and their size ranges from a few dozen to a few hundred micrometres. The pseudo-core-shell powder prepared, as mentioned, maintains that structure during powder manipulation for analysis and further use in producing SPS compacts. It is worth mentioning that the powder consists of core-shell-structured particles and some zinc ferrite fine particles. The free (unattached) zinc ferrite particles help upon densifying to better insulate the particles; surface zinc ferrite discontinuous zones of core-shell particles are better covered.

### 3.2. Permalloy (Supermalloy)@ZnFe_2_O_4_ Compacts Prepared by SPS

**Figure 2:**
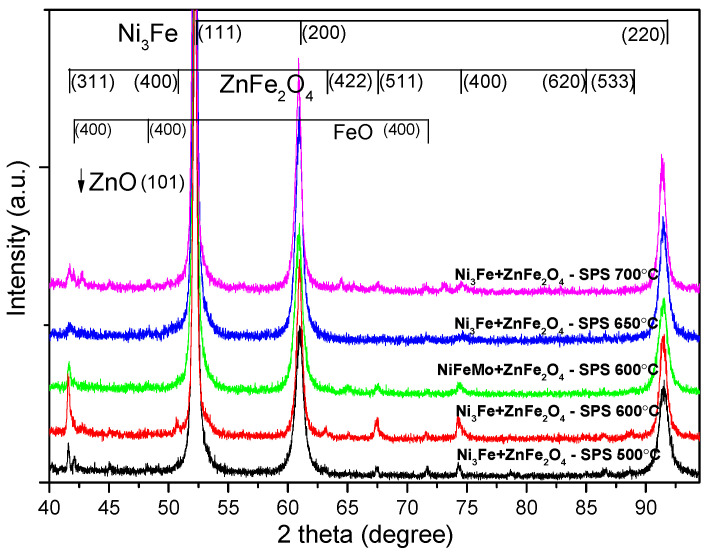
X-ray diffraction patterns for the SPS-ed composite compacts obtained from Ni_3_Fe@ZnFe_2_O_4_ and Supermalloy@ZnFe_2_O_4_ pseudo-core-shell powders as a function of sintering parameters.

The Ni_3_Fe@ZnFe_2_O_4_ and Supermalloy@ZnFe_2_O_4_ pseudo-core-shell powders have been compacted by SPS at 500, 600, 650, and 700 °C without holding time. Figure 2 presents the X-ray diffraction patterns for the SPS-ed compacts produced from Ni_3_Fe@ZnFe_2_O_4_ pseudo-core-shell powders. The Ni_3_Fe FCC structure Bragg peaks (JCPDS file no. 88-1715) are present in the XRD patterns for all the samples. By increasing the sintering temperature, the FWHM of the maxima decreases, thus indicating that the crystallite size increases. The formation of new phases can be observed, as it is indicated by the presence of new diffraction maxima. The newly formed phases that are present in compacts are identified as being FeO (JCPDS file no. 06-0615) and ZnO (JCPDS file no. 36-1451). We can assume that this new phase forms at the interface between the metallic Permalloy and the zinc ferrite older phases by solid-state reactions. These new phases are expected to have a significant influence on the SPS-ed composite compact characteristics. Both phases that are formed upon sintering have high resistivity, and their presence is a positive aspect related to electrical resistivity. As the sintering temperature increases, the intensity of the maxima of the newly formed phases increases, thus indicating the larger amount of these phases in the composite. Indeed, upon increasing the temperature, a higher mobility of the atoms is assured, and the iron atoms diffuse to the outer layer of the metallic particles and react with zinc ferrite, leading to partial decomposition. Actually, since the zinc ferrite formula can also be written as ZnO∙Fe_2_O_3_, an iron atom can react with Fe_2_O_3_, leading to its reduction and thus the formation of FeO.

**Figure 3:**
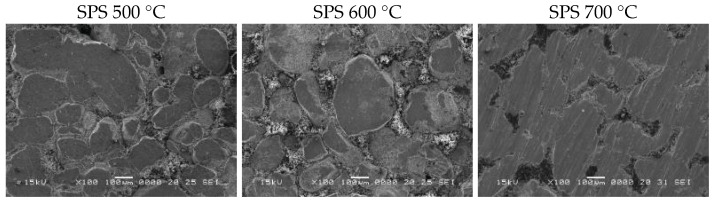
The surface of SPS-ed composite compacts, produced from Ni_3_Fe@ZnFe_2_O_4_ pseudo-core-shell powders. The sintering temperature is shown on top of the SEM images.

The surface of SPS-ed composite compacts, produced from Ni_3_Fe@ZnFe_2_O_4_ pseudo-core-shell powders, are shown in Figure 3. The density of the SPS-ed compacts increases by the sintering temperature increasing. Also, the porosity decreases by increasing the sintering temperature from 500 to 700 °C. The pores are smaller and less visible in the sample sintered at 700 °C compared to the others. The samples’ microstructure consists of metallic zones embedded in an almost continuous matrix/network, which are thinner when the sintering temperature increases. The matrix is connected to the shell formed by Zn ferrite of the Ni_3_Fe@ZnFe_2_O_4_ pseudo-core-shell particles. The particle size has a small variation in the sintering temperature range; our estimation is that the metallic particles have a size of about 100–120 µm. During sintering, some of the metallic particles create sintering necks, which thus results in larger particles.

**Figure 4:**
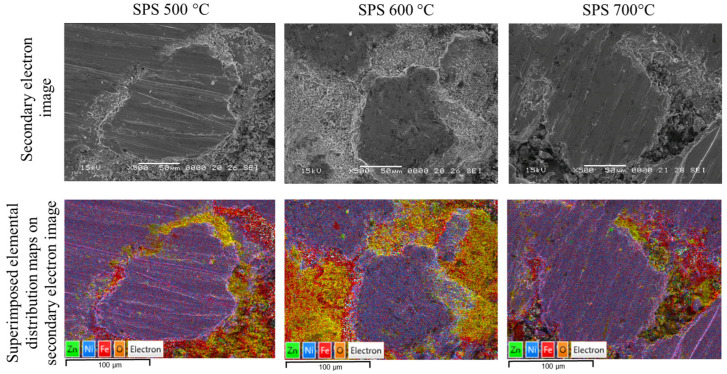
SEM images of SPS-ed and superimposed distribution maps for Ni, Fe, O, and Zn elements in the SPS-ed composite compacts obtained at 500, 600, and 700 °C from Ni_3_Fe@ZnFe_2_O_4_ pseudo-core-shell particles.

In order to evaluate the samples from the point of view of phase distribution, distribution maps of the constituent elements were recorded, following the ferrite distribution in relation to the soft magnetic phase (Ni_3_Fe), Figure 4. The X-ray microanalysis (EDX investigations) confirm the above-described microstructures and phases’ distribution. The nickel-rich zones, provided by the Ni_3_Fe phase, are embedded in a quasi-continuous matrix formed the Fe, O, and Zn elements, provided by zinc ferrite of the Ni_3_Fe@ZnFe_2_O_4_ pseudo-core-shell particles.

**Figure 5:**
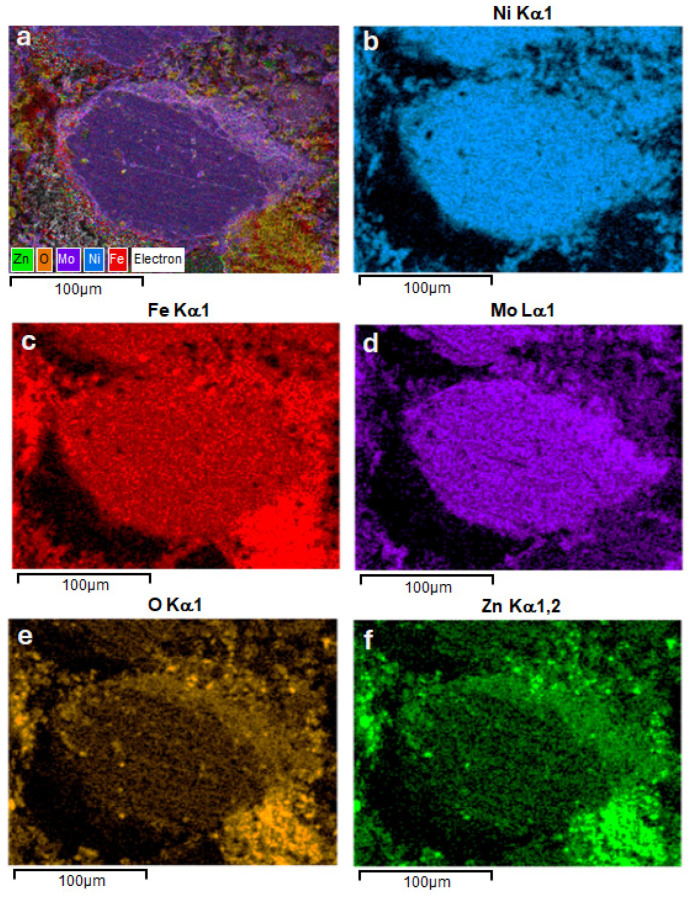
SEM images and distribution maps for Ni, Fe, Mo, O, and Zn elements in the SPS-ed composite compacts produced at 700 °C temperature from Supermalloye@ZnFe_2_O_4_ pseudo-core-shell particles: (**a**)—elemental distribution maps overlapped on the SEM image; (**b**–**f**)—distribution maps for Ni, Fe, O, Mo, and Zn elements, respectively.

The same distribution of the phases (soft magnetic phase—Supermalloy and Zn ferrite) are evidenced also for the SPS-ed compacts produced from Supermalloy@ZnFe_2_O_4_ pseudo-core-shell particles, Figure 5. A SEM image of the compact microstructure is shown in Figure 5a, and in Figure 5b, all the elemental distribution maps that are overlapped on the SEM image from Figure 5a are shown; Figure 5b–f show the individual distribution maps for Ni, Fe, Mo, O, and Zn elements, respectively. Figure 5 confirms that the large Supermalloy particles are embedded in a matrix formed from Zn ferrite particles.

From the point of view of the compactness of the homogenised powders, there are no great variations with temperature. However, for compacts sintered at 700 °C, it can be said that there is a higher compactability compared to the other temperatures. Regardless of the sintering time, it can be observed how the zinc ferrite particles cover the spaces between the Ni_3_Fe. A ferrite distribution at the particle boundary is very promising from the point of view of properties in alternating magnetic fields. In the case of the Supermalloy composite, only the sample sintered at 600 °C (without holding) was analysed. Morphologically, the homogenised powders show a lower density because the fine ferrite particles are at the limit of the large Supermalloy particles. This distribution is also supported by the distribution maps shown in Figure 5.

### 3.3. Density, Electrical, and Magnetic Characteristics of the Permalloy (Supermalloy)@ZnFe_2_O_4_ SMCs

**Figure 6:**
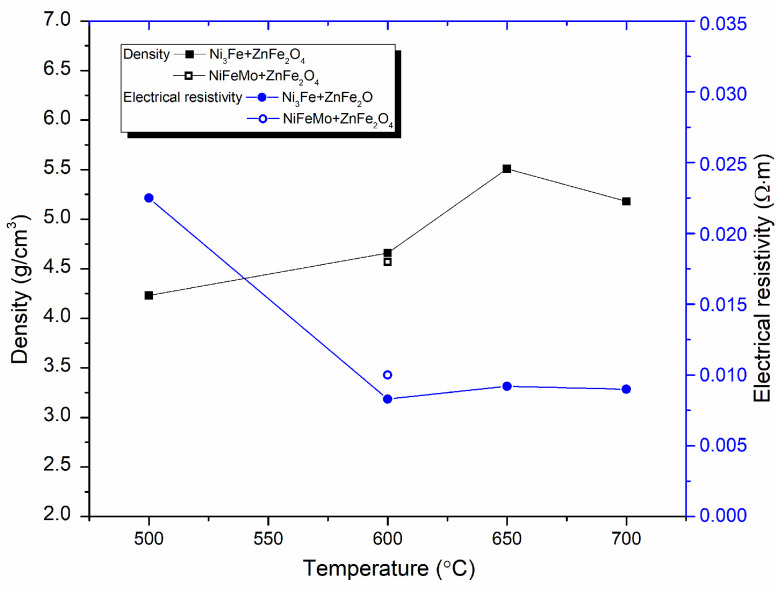
Density and electrical resistivity of plasma sintered Ni_3_Fe/ZnFe_2_O_4_ composite compacts versus temperature.

The influence of sintering temperature on the density and electrical resistivity of the composite compacts produced by SPS from Ni_3_Fe@ZnFe_2_O_4_ and Supermalloy@ZnFe_2_O_4_ pseudo-core-shell powders is shown in Figure 6. 

Figure 6 shows that the density of the composite increases with increasing sintering temperature. In the case of composite compacts produced from pseudo-core-shell powders, Figure 6 shows that the electrical resistivity decreases strongly when the sintering temperature increases from 500 °C to 600 °C, after which it stabilises at values of about 7 × 10^−3^ Ω·m for sintering temperatures between 600–700 °C, in correlation with the increasing density of the sintered compacts. This value is three orders of magnitude higher than the electrical resistivity of sintered Fe compacts (5.9 × 10^−6^ Ω·m), which means that the energy losses in the core due to eddy currents are also three orders of magnitude lower than for magnetic Fe powder cores. 

It is important to note that the electrical resistivity is highly dependent on a lot of factors, such as: (i) the ferrite network continuity, (ii) the sintered compacts density, and (iii) the solid-state reaction between component phases, induced by the sintering process, having result phases with different electrical resistivities.

**Figure 7:**
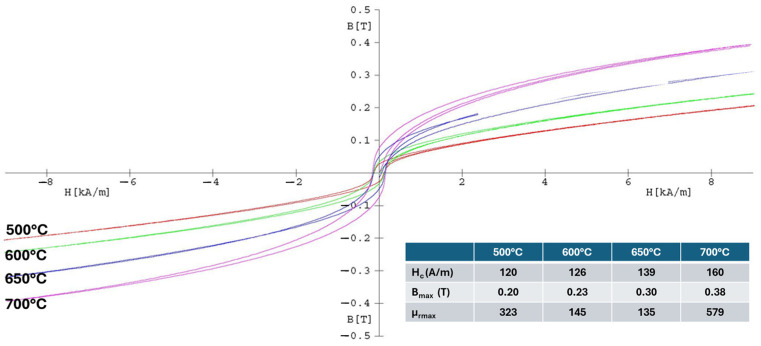
The B–H hysteresis curves in DC of the Ni_3_Fe/ZnFe_2_O_4_ composite compacts, SPS-ed at temperatures: 500, 600, 650, and 700 °C. Holding time was 0 min.

The magnetic properties of the spark plasma sintered composite compacts obtained from the Ni_3_Fe@ZnFe_2_O_4_ and Supermalloy@ZnFe_2_O_4_ pseudo-core-shell powders were studied in DC and in AC mode up to 10,000 Hz. The B–H curves in DC of the Ni_3_Fe/ZnFe_2_O_4_ toroidal compacts obtained by SPS at different sintering temperatures are presented in Figure 7. In the inset table of the figure, the coercive field, the magnetic induction, and the maximum relative permeability are detailed. It can be seen that the magnetic characteristics are better for the compacts produced by sintering at 700 °C, except the coercive field. This is due to the formation of the new phases, ZnO and FeO, besides ZnFe_2_O_4_ and permalloy.

**Figure 8:**
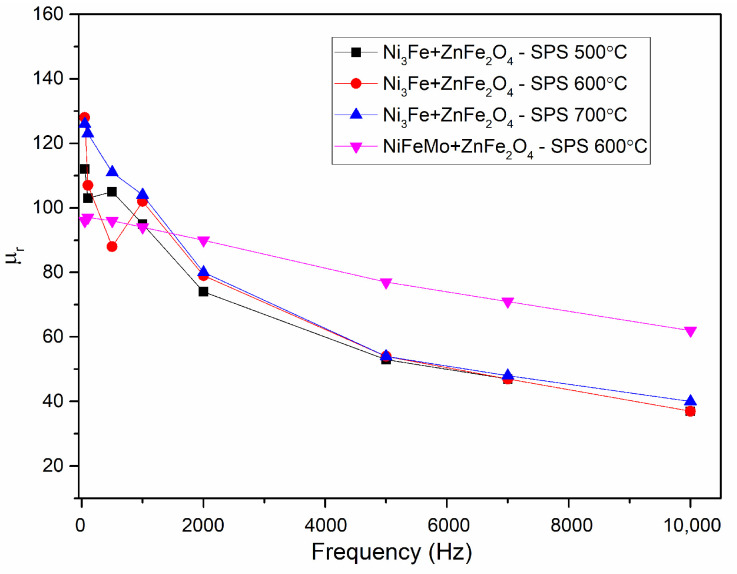
Evolution of initial magnetic permeability vs. frequency for toroidal shaped Ni_3_Fe/ZnFe_2_O_4_ and NiFeMo/ZnFe_2_O_4_ composite compacts, SPS-ed at the sintering temperature indicated on the graph. The induction level was seated at 0.01 T.

Figure 8 presents the evolution of initial magnetic permeability versus frequency for toroidal-shaped Ni_3_Fe/ZnFe_2_O_4_ and NiFeMo/ZnFe_2_O_4_ composite compacts, SPS-ed at the sintering temperature indicated on the graph. The induction level was seated at 0.01 T. 

As it is known, the magnetic permeability is strongly influenced by the porosity, density, and non-magnetic phases of the materials [28,29,30]. A descending permeability evolution is noticed for all the compacts, with the evolution being independent of the sintering temperature. The samples containing Ni_3_Fe show a more rapid decrease, independent of sintering temperature than those containing NiFeMo. A monotony decrease of the relative permeability versus frequency is observed for the NiFeMo/ZnFe_2_O_4_ compact sintered at 700 °C. The higher relative initial permeability was obtained for the Supermalloy/ZnFe_2_O_4_ composite compacts sintered at 600 °C, which decreases linearly for the entire frequency range studied, from around 95 to 50. The relative permeability values are larger than those of the SPS-ed compacts obtained by us from Permalloy (Supermalloy)@Mn_0.5_Zn_0.5_Fe_2_O_4_ pseudo-core-shell particles [24]. The decrease is probably caused by the demagnetising field induced by the development of some eddy currents during increasing frequency. Due to the large electrical resistivity, it is assumed that some intra-particle eddy currents are developing since large particles have been used. The formation of some eddy currents is also suggested by the evolution of the losses upon increasing frequency.

**Figure 9:**
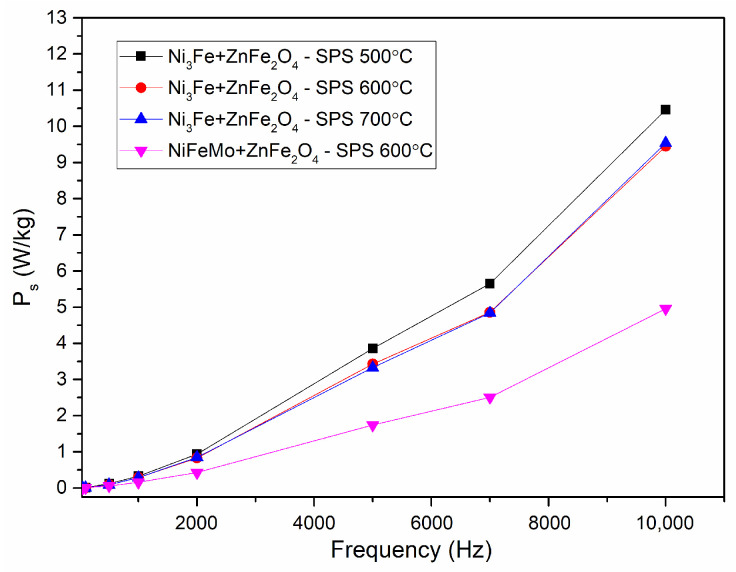
Evolution of the specific power losses vs. frequency for toroidal shaped Ni_3_Fe/ZnFe_2_O_4_ and NiFeMo/ZnFe_2_O_4_ composite compacts; SPS-ed at the sintering temperature is indicated on the graph. The induction level was seated at 0.01 T.

The evolution of the specific power losses versus the frequency for toroidal-shaped Ni_3_Fe/ZnFe_2_O_4_ and NiFeMo/ZnFe_2_O_4_ type composite compacts, obtained at the sintering temperature of 500–700 °C at an induction level of 0.01 T, are shown in Figure 9. A similar evolution of the specific power losses was obtained for all the compacts. Thus, the specific power losses increase exponentially with the increase in the testing frequency. The NiFeMo/ZnFe_2_O_4_ compact SPS-ed at 600 °C has the lowest losses. This can be related to the larger intrinsic electrical resistivity of the Supermaloy as compared to the one of Ni_3_Fe. The results are promising; for the induction level of 0.01 T, until a frequency of 2000 Hz, the power losses are smaller than 1.5 W/kg. At the frequency of 10 kHz, the power losses are larger, but still, they remain at a reduced level, smaller than 15 W/Kg. In the case of Supermalloy/ZnFe_2_O_4_ composite compact SPS-ed at 600 °C, without holding time, the specific power losses are even lower than 5 W/kg. With a certain attribution of the main part of the losses to the eddy current developed in the SPS-ed toroidal samples, mainly at intra-particles level, a decomposition has been performed. The losses deconvolution has been conducted using the Bertotti classical loss separation model [31,32]. The total losses are composed of hysteresis losses and dynamic losses when the excess losses are not considered, as in our case. The dynamic losses are divided in two, inter-particles and intra-particles, as it is well known.

**Figure 10:**
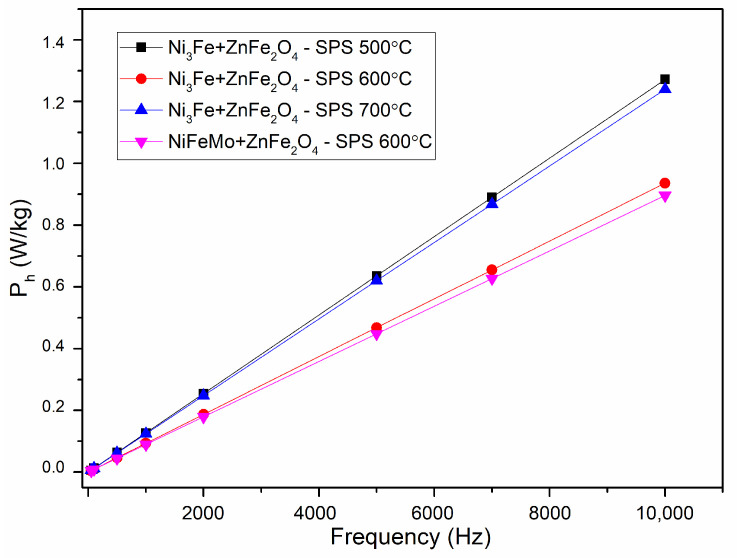
Evolution of the hysteresis losses versus frequency for toroidal shaped Ni_3_Fe/ZnFe_2_O_4_ and NiFeMo/ZnFe_2_O_4_ composite compacts; SPS-ed at the temperature is indicated on the graph. The induction level was seated at 0.01 T.

The evolution of the hysteresis losses versus frequency at 0.01 T are presented in Figure 10. It can be noticed that the lower losses are given by the samples sintered at 600 °C. This is related to the samples’ density and composite component phases. For these samples, the sintering temperature is limiting the formation of multiple phases at the interface.

**Figure 11:**
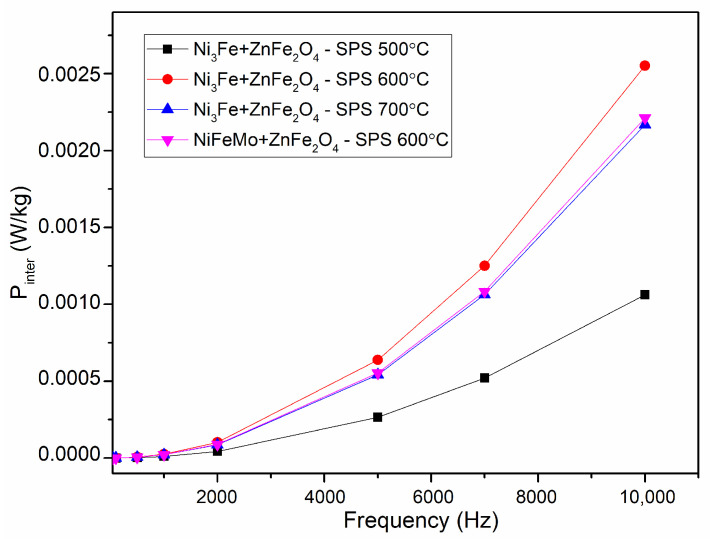
Evolution of the inter-particles losses versus frequency for toroidal shaped Ni_3_Fe/ZnFe_2_O_4_ and NiFeMo/ZnFe_2_O_4_ composite compacts; SPS-ed at the temperature is indicated on the graph. The induction level was seated at 0.01 T.

The inter-particles losses evolution is given in Figure 11. A low value can be noticed for all the SMCs, which confirms the good insulation of the ferromagnetic particles in the sintered compacts. The evolution of the inter-particles losses is correlated with the sample’s density and electrical resistivity. The inter-particles losses are lower compared to the hysteresis losses.

**Figure 12:**
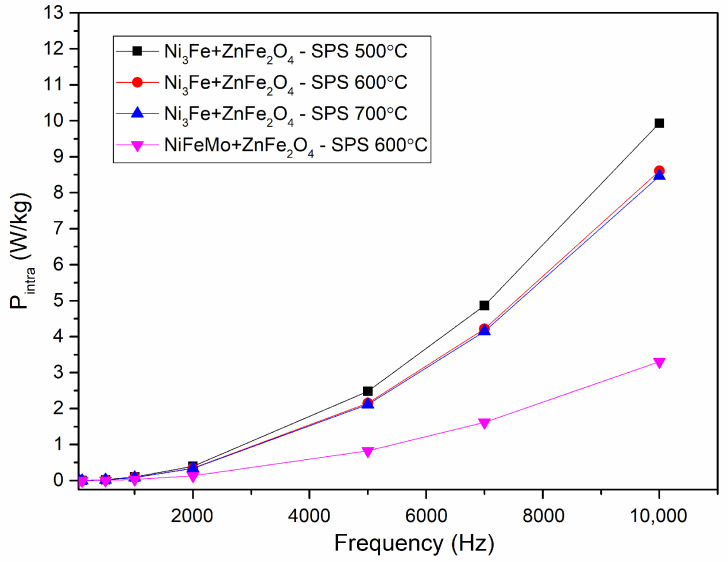
Evolution of the intra-particles losses versus frequency for toroidal shaped Ni_3_Fe/ZnFe_2_O_4_ and NiFeMo/ZnFe_2_O_4_ composite compacts; SPS-ed is at the temperature indicated on the graph. The induction level was seated at 0.01 T.

The intra-particles losses are depicted in Figure 12. It can be observed that these losses represent the main part of the losses. The lower intra-particles losses are obtained for the samples containing NiFeMo. This is related to the higher intrinsic electrical resistivity of this alloy compared to Ni_3_Fe. The use of small particles is suggested and thus will lead to lower intra-particles losses. Also, it can be noticed that the formation of FeO and ZnO in a higher amount has a positive effect on diminishing the intra-particles losses. This is related to the reduction of effective particles size by the reaction on their surface of the iron (provided by Permalloy and Supermalloy) with zinc ferrite. The amount of FeO and ZnO are certainly at a relatively high amount, their maxima are easily visible in diffractograms, and the intensity increases upon increasing the sintering temperature.

## 4. Conclusions

Composite particles with the pseudo-core-shell structure of Ni_3_Fe@ZnFe_2_O_4_ and NiFeMo/ZnFe_2_O_4_ types have been successfully produced from large ferromagnetic particles (Ni_3_Fe and NiFeMo) covered by a layer of fine Zn ferrite by mixing and homogenising for 15 min in a Turbula-type spatial homogeniser. 

The pseudo-core-shell structure of the powder has been highlighted by SEM and EDX studies. The composite powder has been subjected to spark plasma sintering in the temperature range of 500 to 700 °C. Soft magnetic composites with Ni-based particles embedded in an oxide matrix/network consisting mainly of ZnFe_2_O_4_ alongside the FeO and ZnO have been obtained by this technique. ZnO and FeO are formed upon sintering by the reaction of iron atoms provided by Permalloy/Supermalloy and zinc ferrite at the interface of core-shell composite particles. The formation of the zinc and iron oxides has a positive effect on the powder densification and also on the compact’s electrical resistivity. The tests of the soft magnetic composite in the AC mode confirmed that the compacts have a structure that has a high electrical resistivity which limits/diminishes the development of the eddy currents at an interparticle level. This was clearly demonstrated by the power losses separation, which indicated that the main part of the losses is given by intra-particles losses due to the use of large ferromagnetic particles. The use of ferromagnetic particles with lower sizes will ensure the decrease of the intra-particles losses.

## Data Availability

The authors confirm that the data supporting the findings of this study are available within the article.

## References

[1] Shokrollahi H., Janghorban K. (2007). Soft magnetic composite materials (SMCs). J. Mater. Process. Technol..

[2] Silveyra J.M., Ferrara E., Huber D.L., Monson T.C. (2018). Soft magnetic materials for a sustainable and electrified world. Science.

[3] Fan X., Wang J., Wu Z., Li G. (2015). Core–shell structured FeSiAl/SiO2 particles and Fe3Si/Al2O3 soft magnetic composite cores with tunable insulating layer thicknesses. Mater. Sci. Eng. B.

[4] Périgo E.A., Weidenfeller B., Kollár P., Füzer J. (2018). Past, present, and future of soft magnetic composites. Appl. Phys. Rev..

[5] Sunday K.J., Taheri M. (2017). Soft magnetic composites: Recent advancements in the technology. Met. Powder Rep..

[6] Streckova M., Szabo J., Batko I., Batkova M., Bircakova Z., Fuzer J., Kollar P., Kovalcikova A., Bures R., Medvecky L. (2020). Design of Permalloy–ferrite–polymer soft magnetic composites doped by ferrite nanoparticles and visualisation of magnetic domains. Bull. Mater. Sci..

[7] Calata J.N., Lu G.Q., Ngo K. (2014). Soft Magnetic Alloy–Polymer Composite for High-Frequency Power Electronics Application. J. Electron. Mater..

[8] Kordecki A., Weglinski B. (1990). Development and applications of soft magnetic PM materials. Powder Metall..

[9] Weglinski B., Jenkins J., Wood J.V. (1991). Soft Magnetic PM Materials in Selected Case Studies in Powder Metallurgy.

[10] Kordecki A., Weglinski B. (1988). Dielectromagnetics Containing Different Dielectrics. Powder Metall..

[11] Neamţu B.V., Geoffroy O., Chicinaş I., Isnard O. (2012). AC magnetic properties of the soft magnetic composites based on Supermalloy nanocrystalline powder prepared by mechanical alloying. Mater. Sci. Eng. B.

[12] Hirota K., Kato M., Taguchi H. (2011). Fabrication of full-density Mg-ferrite/Fe-Ni permalloy nanocomposites with a hight-saturation magnetisation density of 1T. Int. J. Appl. Ceram. Technol..

[13] Moulin J., Champion Y., Varga L.K. (2002). Magnetic properties of nanocomposites containing Fe-Ni or Fe dispered in a Mn-Zn ferrite matrix. IEEE Trans. Magn..

[14] Fang M., Strom V., Olsson R.T., Rao K.V. (2012). Particle size and magnetic properties dependence on grow temperature for srapid mixed co-precipitated magnetite nanoparticles. Nanotechnology.

[15] Wilson D., Langell M.A. (2014). XPS analysis of oleylamine/oleic acid capped Fe_3_O_4_ nanoparticles as a function of temperature. Appl. Surf. Sci..

[16] Kalarus J., Kogias G., Holz D., Zaspalis V.T. (2012). High permeability–high frequency stable MnZn ferrites. J. Magn. Magn. Mater..

[17] Akther Hossain A.K.M., Mahmud S.T., Seki M., Kawaib T., Tabata H. (2007). Structural, electrical transport, and magnetic properties of Ni1−xZnxFe_2_O_4_. J. Magn. Magn. Mater..

[18] Tokita M. (2013). Spark Plasma Sintering (SPS) Method, Systems, and Applications in Shigeyuki Somiya. Handbook of Advanced Ceramics.

[19] Wang L., Zhang J., Jiang W. (2013). Recent development in reactive synthesis of nanostructured bulk materials by spark plasma sintering. Int. J. Refract. Met. Hard Mater..

[20] Wang M., Zan Z., Deng N., Zhao Z. (2014). Preparation of pure iron/Ni–Zn ferrite high strength soft magnetic composite by spark plasma sintering. J. Magn. Magn. Mater..

[21] Hu Z.Y., Zhang Z.H., Cheng X.W., Wang F.C., Zhang Y.F., Li S.L. (2020). A review of multi-physical fields induced phenomena and effects in spark plasma sintering: Fundamentals and applications. Mater. Des..

[22] Marinca T.F., Popa F., Neamțu B.V., Prică C.V., Chicinaș I. (2023). Permalloy/alumina soft magnetic composite compacts obtained by reaction of Al-permalloy with Fe_2_O_3_ nanoparticles upon spark plasma sintering. Ceram. Int..

[23] Marinca T.F., Cotojman L., Neamțu B.V., Popa F., Prică C.V., Hirian R., Sechel N.A., Ciascai I., Chicinaș I. (2024). Soft magnetic composite of Ni_3_Fe/ZnFe_2_O_4_ type obtained by mechanical alloying/milling and spark plasma sintering. Ceram. Int..

[24] Cotojman L., Marinca T.F., Popa F., Neamțu B.V., Prică V.C., Chicinaș I. (2023). Producing Soft Magnetic Composites by Spark Plasma Sintering of Pseudo Core–Shell Ni–Fe Alloy@Mn_0_._5_Zn_0_._5_Fe_2_O_4_ Powders. Materials.

[25] Chicinaș I., Pop V., Isnard O. (2004). Synthesis of the Supermalloy powders by mechanical alloying. J. Mater. Sci..

[26] Popa F., Isnard O., Chicinaș I., Pop V. (2010). Synthesis of nanocrystalline Supermalloy powders by mechanical alloying: A thermomagnetic analysis. J. Magn. Magn. Mater..

[27] Neamţu B.V., Chicinaş I., Isnard O., Popa F., Pop V. (2011). Influence of wet milling conditions on the structural and magnetic properties of Ni_3_Fe nanocrystalline intermetallic compound. Intermetallics.

[28] Onderko F., Birčáková Z., Dobák S., Kollár P., Tkáč M., Fáberová M., Füzer J., Bureš R., Szabó J. (2022). Magnetic properties of soft magnetic Fe@SiO_2_/ferrite composites prepared by wet/dry method. J. Magn. Magn. Mater..

[29] Bozorth R.M. (1993). Ferromagnetism.

[30] Cullity B.D., Graham C.D. (2009). Introduction to Magnetic Materials.

[31] Bertotti G. (1988). General properties of power losses in soft ferromagnetic materials. IEEE Trans. Magn..

[32] Kollár P., Olekšáková D., Vojtek V., Füzer J., Fáberová M., Bureš R. (2017). Steinmetz law for acmagnetised iron-phenolformaldehyde resin soft magnetic composites. J. Magn. Magn. Mater..

